# Not All Flavor Expertise Is Equal: The Language of Wine and Coffee Experts

**DOI:** 10.1371/journal.pone.0155845

**Published:** 2016-06-20

**Authors:** Ilja Croijmans, Asifa Majid

**Affiliations:** 1 Centre for Language Studies, Radboud University, Nijmegen, The Netherlands; 2 International Max Planck Research School for Language Sciences, Nijmegen, The Netherlands; 3 Donders Institute for Brain, Cognition, and Behaviour, Radboud University, Nijmegen, The Netherlands; 4 Max Planck Institute for Psycholinguistics, Nijmegen, The Netherlands; Duke University Medical Center, UNITED STATES

## Abstract

People in Western cultures are poor at naming smells and flavors. However, for wine and coffee experts, describing smells and flavors is part of their daily routine. So are experts better than lay people at conveying smells and flavors in language? If smells and flavors are more easily linguistically expressed by experts, or more “codable”, then experts should be better than novices at describing smells and flavors. If experts are indeed better, we can also ask how general this advantage is: do experts show higher codability only for smells and flavors they are expert in (i.e., wine experts for wine and coffee experts for coffee) or is their linguistic dexterity more general? To address these questions, wine experts, coffee experts, and novices were asked to describe the smell and flavor of wines, coffees, everyday odors, and basic tastes. The resulting descriptions were compared on a number of measures. We found expertise endows a modest advantage in smell and flavor naming. Wine experts showed more consistency in how they described wine smells and flavors than coffee experts, and novices; but coffee experts were not more consistent for coffee descriptions. Neither expert group was any more accurate at identifying everyday smells or tastes. Interestingly, both wine and coffee experts tended to use more source-based terms (e.g., *vanilla*) in descriptions of their own area of expertise whereas novices tended to use more evaluative terms (e.g., *nice*). However, the overall linguistic strategies for both groups were en par. To conclude, experts only have a limited, domain-specific advantage when communicating about smells and flavors. The ability to communicate about smells and flavors is a matter not only of perceptual training, but specific linguistic training too.

## Introduction

Wine, coffee, cheese, and chocolate would all taste bland without the sense of smell. Even though smells are omnipresent in our daily lives, people struggle with odor and flavor naming (i.e., the multisensory experience in the mouth including gustatory, olfactory, and somatosensory sensations; [1,2]). If asked to name everyday odors, like peanut butter, cinnamon or strawberry, most people can only name half of them correctly [3–6].

At the same time, there is a lucrative industry around language and flavor. Influential wine experts have considerable impact on the price and sales of a wine just through their reviews [7]. This is an interesting state of affairs, as some wine authors themselves acknowledge the limits of language when describing smells and flavors [8–10].

English, like other Western languages, appears to have a restricted vocabulary for smells and tastes [11,12]. A simple comparison of the brute number of terms for the senses leaves smell and taste at the bottom of the hierarchy [13,14]. When English speakers do try to name smells and flavors they overwhelmingly rely on source-descriptions (e.g., *it smells like a banana; it tastes like chicken)* or metaphors (e.g., *it smells green; it tastes wicked)*. Furthermore, English speakers show low accuracy, consistency and agreement in how they describe smells and flavors (e.g. [15–19]).

Recently the universality of these findings has been questioned [20,21]. For example, Jahai [21,22] and Maniq [23], two Aslian languages spoken in the Malay Peninsula by hunting-gathering communities, have dedicated vocabulary for smells. The smell of different perfumes, flowers, durian and bearcat (*Arctitis binturong)* is described by the Jahai as *ltpɨt*, whereas Maniq might describe the smell of some food (e.g., tubers), bearcat, clean clothes, and some trees with *lspəs* [22,23]. Majid and Burenhult [21] also found Jahai speakers name odors as easily as colors, unlike English speakers who struggled to name the same odors. This raises the possibility that the difficulty people have in naming smells and flavors could be a WEIRD (Western, Educated, Industrialized, Rich, Democratic [24]) affair.

Odors play an important role in Jahai daily life. This is reflected not only in language, but in various aspects of Jahai culture, such as religion and medicine [22]. According to the Jahai, some types of illness are cured by healing magic involving fragrant smells from plants and burnt resins, for example. Similarly, personal names are often drawn from the names of fragrant plants and flowers. For the Jahai, a cultural preoccupation with odors, therefore, aligns with their dexterity in talking about smells.

In the West, naming odors and flavors is also important for some people. Like perfumers, wine experts have years of training and experience in appreciating and describing odors, as well as flavors [25]. This is illustrated by “tastings”, during which experts describe and discuss wines, and compare notes. So wine experts can be considered to be part of a distinct sub-culture with its own communicative practices and rituals around smells and flavors (cf. [26]). Considering the significance of flavor in their occupation, then, are wine experts, or other flavor experts, better at describing smells and flavors than novices? And, if so, what linguistic strategies do they use? The previous literature shows no general agreement on these matters, as described below.

### The language of wine experts

Wine is a complex entity, with as many as 800 different aromatic volatiles that together create a high dimensional flavor experience [27]. How do wine experts and novices convey their personal wine experience to each other given this complexity?

Cain [3] has suggested wine experts appreciate flavors in a different way than novices. A casual perusal of wine reviews certainly adds to this impression. Consider this tasting note:

*The 2001 Batard-Montrachet offers a thick*, *dense aromatic profile of toasted white and yellow fruits*. *This rich*, *corpulent offering reveals lush layers of chewy buttered popcorn flavors*. *Medium-bodied and extroverted*, *this is a street-walker of a wine*, *making up for its lack of class and refinement with its well-rounded*, *sexually-charged assets*. *Projected maturity*: *now-2009*. ([28] p. 57)

As Suarez-Toste [28] notes, this description contains many figurative and metaphorical constructions. Metaphors are ubiquitous in experts’ wine descriptions [28–31]: wines are described as having a body (e.g., ‘this rich, corpulent offering’ [28]) and persona (e.g. ‘making up for its lack of class and refinement’ [28]). Wines are also described as if they were animate, and capable of motion (e.g., ‘This wine bursts from the glass with violets’ [32]).

So, it seems as if wine experts are vague and literary in their descriptions. However, other studies suggest experts use more concrete words (e.g., *blackberries* instead of *fruity*; [33–36]), and provide more precise labels (e.g., *gooseberry* instead of *fruit* [37]). It has also been suggested experts use more wine-domain specific terminology (e.g., *metallic*, *mineral*, *unripe* [38,39]), more technical terms (e.g., *aldehyde*), and make less reference to hedonic value (e.g., *unpleasant* [40]). Thus, there is contradictory evidence about the types of strategies experts use to convey their experiences.

Turning to whether experts have more communicative success than novices, the jury is also out. On the one hand, there are studies suggesting wine experts might have an advantage over novices in how they communicate about wines. Wine experts appear to agree with each other more about how to name wine-related odors than novices or intermediate wine students [37,41–43]. Some studies have also found expert descriptions are more often matched to the correct wine than descriptions composed by novices [34,35,44]. This fits with the idea proposed by Smith [45] that experts agree more on the smell and flavor of wine, given their shared experiences.

On the other hand, other studies suggest experts are not better at describing flavors than novices. For example, Lawless [34] compared expert wine descriptions to those of novices, and found expert descriptions were highly idiosyncratic, with most terms used only once by one participant. This suggests there is little systematicity between experts. In another study, experts showed similar levels of agreement as novices in their descriptions of wine-related odors [46]. However these studies can be interpreted in a different way. Lawless [34] did not directly compare the two groups on consistency, so we cannot be sure whether experts and novices were similar or different on this measure. Similarly, a closer look at the data in Parr et al. [46] shows experts had numerically higher identification and consistency rates than novices, leaving open the possibility the study was underpowered (as suggested by the authors also, on p. 752). Overall, the few studies conducted to date contradict each other, and leave open the question of whether experts are better at naming odors and flavors.

### How general is expertise?

If wine experts are indeed better at naming odors and flavors, this leads to the question of how well odor naming in one domain generalizes to another. That is, if there is an odor naming advantage for wine experts, does it hold for odors outside of their domain of expertise? Zucco and colleagues [37] found wine experts were better at naming odors than intermediate wine students, but this advantage was restricted to wine-related odors only, and did not extend to household odors. A more recent study [40] compared the language different experts (flavorists and perfumers) used to describe common odors. Flavorists and perfumers used different words than novices, but they found no difference between expert groups, which could indicate flavor experts possess a general ability to express smells and flavors in language.

Sezille et al. [40] are unusual in comparing flavorists and perfumers. Most previous studies focus exclusively on wine experts, and compare them to novices (for a recent review, see [47]). In fact, there are many expert domains which would make for an interesting comparison to wine. Take coffee, for example. Just like wine, coffee contains more than 800 volatile aroma components (cf. [48,49]). There is an extensive literature regarding the growth, harvest, processing, production, and marketing of both wines and coffees. In addition, experts in both domains typically undergo extensive training: it takes many years of experience to become an expert in either specialty.

Nevertheless, coffee and wine expertise also differs in some interesting respects. Whereas wines are usually elaborately described in tasting notes, menus, and on placards in stores, the descriptions of coffees tend to be less frequently encountered. This can be quantified further in a number of ways. For example, there are at least 10 different subscription magazines to be found about wine on Amazon.com, but not a single one for coffee (retrieved December 1^st^ 2015). A simple Google search on both topics reveals a similar asymmetry: a Dutch query for wine tasting notes (*“wijn” AND “proefnotitie”*) returned 77,000 web pages containing wine tasting notes, while a similar query for coffee (*“koffie” AND “proefnotitie”*) returned a mere 10,000 web pages containing coffee tasting notes (retrieved October 16^th^ 2015). The same query in English revealed a similar picture: 501,000 results for wine tasting notes (“*wine” AND “tasting note”*) versus only 81,000 for coffee tasting notes (“*coffee” AND “tasting note”*, retrieved December 8^th^ 2015). Likewise, any reasonably priced restaurant will provide a written description of wines on the menu; most supermarkets provide additional information about the wines they sell. But comparably detailed descriptions of coffees are rare. This asymmetry could be attributed to the number of wine vs. coffee experts, but this still could have relevance for sensory language. Studies demonstrate more exposure to more varied input from different people can influence language use (e.g. [50]). For this reason, in this study we compared coffee experts to wine experts on the same flavor and odor naming tasks. If domain-specific linguistic experience matters, then wine and coffee experts should behave differently because there are more (in number) and more varied (number of people producing) descriptions for wines than coffees.

The question we asked is whether smells and flavors are linguistically expressed more easily by wine and coffee experts than by novices. Are they more “codable”? Items that are more codable in language have (1) shorter lengths; (2) dedicated vocabulary for their expression; and (3a) are named more consistently and (3b) correctly (cf. [21,51]). We tested whether experts and novices differ on these measures in how they describe smells and flavors.

If the chemical senses are easier to communicate about for experts who have perceptual expertise and training in smells and flavors, like the wine and coffee experts in this study, then smells and flavors should be more linguistically codable for them than they are for novices. And this should be true regardless of the specific smells and flavors. That is, if wine or coffee expertise is equivalent to the kind of “expertise” the hunting-gathering Jahai have, then experts should be better at describing smells (and flavors) regardless of the source. If, on the other hand, expertise is limited, i.e., experts only have domain-specific expertise, then wine experts should show higher codability for wines; coffee experts for coffee; and neither group should differ from each other, or the novices, on basic odors and tastes. Finally, if the kind of language games around expertise is important (e.g., how often people write and talk about their domain of expertise), we might expect wine experts to show higher codability than coffee experts, because they engage in discussions over their specialty more often and receive more varied input.

## Methods

### Ethics statement

Each participant was informed about the purpose and methods of the study, and written consent was obtained before the experiment began. The study was approved by the institutional Ethics Assessment Committee of Radboud University.

### Participants

Sixty-three participants (22 women, *M*_age_ = 43.7 years, *SD* = 11.7, age range: 24–70 years) including wine experts, coffee experts, and novices participated in the experiment (see Table 1). Participants were actively recruited by approaching experts in stores, word-of-mouth, via websites and e-mail, and social media. Participants were not paid, but were reimbursed for travel as appropriate.

**Table 1:** Participant characteristics.

	Wine experts	Coffee experts	Novices
Number	22	20	21
Gender (number of women)	7	8	7
Mean age	45.8	38.9	45.9
Age Range	29–61	26–52	24–70

All participants were native speakers of Dutch, except for one wine expert, who moved from France to the Netherlands at a young age and spoke Dutch at near-native level. They were otherwise relatively homogenous. Wine experts had a vinologist degree and/or worked as a qualified, experienced vinologist or sommelier (cf. [39,46]). Coffee experts worked as qualified baristas, coffee roasters, or coffee brokers. The only criterion for novices was consumption of at least one glass of wine and one cup of coffee per week, to ensure they were familiar with the smell and flavor of both. In fact, the groups differed in wine and coffee consumption. Wine experts consumed significantly more wine than coffee experts or novices, χ^2^ (6, *N* = 65) = 24.0, *p* = .001, Cramer’s V = .43, while coffee experts consumed more coffee than wine experts or novices, χ^2^ (6, *N* = 65) = 12.3, *p* = 0.056, Cramer’s V = .31.

To validate the expertise levels of the wine and coffee experts, each participant completed three questionnaires: the Wine Knowledge Test [38,39,52], Coffee Knowledge Test (constructed in analogy to the Wine Knowledge Test), and a shortened version of the Odor Awareness Scale [53].

There was a significant difference between groups on the Wine Knowledge Test *F*(2, 60) = 75.24, *p* < .001, η^2^ = .71. Pairwise comparisons showed wine experts had significantly higher scores (*M* = 6.6, *SD* = 1.0) than coffee experts (*M* = 2.5, *SD* = 1.2), *p* < .001, *d* = 3.71 (Bonferroni correction is applied to pairwise comparisons throughout as appropriate), and novices (*M* = 3.0, *SD* = 1.4), *p* < .001, *d* = 2.96; while coffee experts and novices did not differ from each other *p* = .551. Similarly, the groups differed on the Coffee Knowledge Test *F*(2, 59) = 36.34, *p* < .001, η^2^ = .59. Coffee experts had significantly more coffee knowledge (*M* = 6.1, *SD* = 1.7) than wine experts (*M* = 2.7, *SD* = 1.2), *p* < .001, *d* = 2.31, and novices (*M* = 2.8, *SD* = 1.5), *p* < .001, *d* = 2.06; whereas scores of novices and wine experts did not differ, *p* = .694. Finally, the scores of the Odor Awareness Scale also differed across groups *F*(2, 59) = 9.07, *p =* .001, η^2^ = .24: Novices had significantly lower scores (*M* = 23.9, *SD* = 9.2) than wine experts (*M* = 31.6, *SD* = 8.3), *p* = .001, *d* = .88, and coffee experts (*M* = 30.3, *SD* = 5.7), *p =* .030, *d* = .84, but both expert groups were equally aware of their sense of smell in daily life, *p* = .460. This further confirms olfaction is more important for both expert groups than the ordinary person.

### Materials

#### Wines

The five red wines originated from different countries, had different vinification styles, and were chosen for their distinct flavor profiles (in consultation with a vinologist who did not participate in the study; see Table 2). The bottles were opened at least 20 minutes before each testing session, checked for faults (e.g., corkstain), kept at room temperature (20 ± 2°C) in between sessions, and were kept refrigerated overnight. New bottles were opened every three days. Approximately 50 ml of each wine was poured in numbered, transparent crystal wine glasses with a volume of 400 ml.

**Table 2:** Wines and coffees used in the study.

Wine	Name	Country of production	Coffee	Name	Country of production
1	Jean Bousquet Malbec	Argentina	1	Santa Helena Caturra	Colombia
2	Zenato Valpolicella Superiore	Italy	2	Kirimiro Red Bourbon	Burundi
3	Altos R Rioja Temperanillo	Spain	3	Knots Family Heirloom varietals	Ethiopia
4	Vallon des Sources Vacqueyras	France	4	Fazenda Rainha Yellow Bourbon	Brazil
5	Castello de Molina Cabernet Sauvignon	Chile	5	Hacienda Sonora Villa Sarchï	Costa Rica

#### Coffees

Five types of coffee beans from different countries with single estate origin were chosen for their distinct flavor profiles, in analogy with the selected wines (Table 2). These were selected in consultation with a Specialty Coffee Association Europe (SCAE) certified coffee roaster who did not participate in the study. The coffees were roasted in the same way in one batch. Immediately after roasting, the beans were sealed in dark aluminum coated plastic bags, in small lots of 100 grams. To ensure freshness of the coffee, at most three hours prior to testing 13.5 grams of each coffee was weighed and ground medium-fine. New sealed bags of coffee were opened every three days. The experimenter was trained by an independent SCAE barista to prepare the coffee following the Specialty Coffee Association America (SCAA) guidelines for cupping [54]. The coffees were presented in double-walled transparent cups of 250 ml and covered with numbered porcelain saucers until preparation.

#### Comparability of wine and coffee stimuli

As stated, wines and coffees were chosen to be equally distinct from one another. To verify whether the relative perceptual differences between wines and coffees were comparable, a separate experiment was conducted. Twenty naïve participants (13 women, *M*_age_ = 24 years, *SD* = 4.8, age range = 18–38) were asked to sort the five wines and five coffees based on how similar they were to one another. Half the participants sorted wines first; half coffee first. Participants indicated similarity by placing the glasses containing the drink on an A2 (42x49 cm) sheet of paper. The closer 2 stimuli were placed next to each other, the more similar the participant deemed them to be. The *x-* and *y-*coordinates of each stimulus were recorded in millimeters and transformed into interstimulus distances for each stimulus pair.

The mean distance for wines (*M* = 254, *SD =* 53) was not significantly different to the mean distance between coffees (*M* = 237, *SD =* 55) across participants *t*(19) = 1.88, *p* = .074, indicating wines and coffees were comparably perceptually different to each other. There was also a significant correlation between the relative distances between wines and coffees, *r*(18) = .703, *p* < .001, so if a participant sorted wines with a small interstimulus distance, they sorted the coffees in a similar way.

To further explore the perceptual space the wines and coffees occupied, two separate Multiple Factor Analyses were performed using the R package *FactoMineR* [55,56]. For both stimulus types, the data was best fitted with a maximal, four-dimensional solution, with eigenvalues for the four dimensions explaining respectively 42.8%, 23.3%, 18.3%, and 15.6% of the variance for the wines, and 38.8%, 25.6%, 19.6%, and 15.9% of the variance for coffee. This also points to the relative perceptual comparability of the two stimulus sets.

#### Odor stimuli

Participants had to name ten different odors. The odors were presented using Sniffin’ Sticks [57], and were a mixture of edible and inedible objects, covering the pleasantness continuum. The odors were lemon, apple, garlic, rose, chocolate, clove, mushroom, grass, leather, and cinnamon.

#### Taste stimuli

A total of eight taste solutions, sweet, salty, bitter and sour, in strong and weak concentrations, were prepared. Refined sugar (10 grams, 292mM, *sucrose*), salt (7.5 grams, 1283mM, *sodium chloride*), quinine (0.05 grams, 1.54mM, *quinine hydrochloride*) and citric acid (5 grams, 237mM) were dissolved in 100 ml of filtered, boiled water to make strong solutions. Weak solutions were half the concentration [17,18,58,59].

### Procedure

Participants started naming either the wines or coffees first (order counterbalanced). For wines, participants were instructed to first smell and taste each wine, without talking, to familiarize themselves with the stimuli. The participant was then asked: ‘Could you smell the first wine and describe the smell as precisely as possible?’ (in Dutch: *Wilt u nu de eerste wijn ruiken en de geur zo precies mogelijk beschrijven?*). After describing the smell, the participant was asked: ‘Could you now taste the wine and describe the flavor as precisely as possible?’ (*Wilt u nu de wijn proeven en de smaak zo precies mogelijk beschrijven?*). They then moved to the next stimulus until complete. The coffee flavor naming task was the same, with a familiarization phase, followed by describing the smells and then the flavors.

After the wine and coffee naming tasks, participants completed the two expertise questionnaires and odor awareness questionnaire, and then participated in the odor and taste naming tasks. For the odor naming task, each odor pen was uncapped by the experimenter and handed to the participant with the instruction: ‘Can you describe the smell as precisely as possible?’ (*Kunt u de geur zo precies mogelijk beschrijven?*). For the taste naming task, participants were first warned some of the sprays might taste unpleasant. The participants were instructed: ‘Could you now spray the taste on your tongue, and describe what you taste?’ (*Wilt u nu de smaak op uw tong sprayen*, *en beschrijven wat u proeft?*). Participants were allowed to spray the tastant a second time if they wished. After each taste, participants drank some filtered water. All stimuli were presented in a fixed order within each block, and there was a delay of at least 20 seconds between them (following [60]). In practice, the interstimulus interval was between 30 and 35 seconds. The sessions took place in a well-lit, well-ventilated room. All answers were recorded using an audio-recorder.

### Data processing

Audio-recordings were transcribed, and coded separately for the smell and flavor of wine and coffee, the smell of odor stimuli, and taste of basic tastants. To recap, things that are codable in language should be (1) concise; (2) have dedicated terminology; (3a) be described consistently and (3b) correctly. We operationalized each of these measures as follows:

First, the length of the description was measured by counting the number of characters in the fully transcribed response. Short descriptions would indicate higher codability than longer descriptions.

Second, we coded the types of responses participants gave in order to test whether experts differed from novices in the strategies they used to describe smells and flavors. Three categories were identified: (1) Source-based terms, i.e., words referring to objects that could emit that odor or flavor, e.g. *kersen* ‘cherries’, *fruitig* ‘fruity’; (2) Evaluative terms, i.e., words describing hedonic evaluation, e.g., *lekker* ‘pleasant’, *mooi* ‘nice’, *gadverdamme* ‘disgusting’, and (3) Non-source-based terms, i.e., words not referring directly to an object. This latter category is included following Majid and Burenhult [21] who identified a third category of abstract or “basic” terms. In Dutch this includes terms such as *aromatisch* ‘fragrant/aromatic’ and *muf* ‘musty’. Participants rarely used this strategy; however, they did use other non-source-based descriptions such as cross-modal metaphors (e.g., *zoet* ‘sweet’, *bitter* ‘bitter’, *groen* ‘green’), reference to a general state (e.g., *gekookt* ‘cooked’), or associations with events or situations (e.g., *winters* ‘wintery’, *bij de slager* ‘at the butcher’). We could, therefore, test whether experts and novices differed in the extent to which they gave evaluations, referred to a concrete source, or gave more abstract non-source-based descriptions.

Finally, we measured if speakers agreed in how they described smells and flavors. One way to operationalize this is in terms of naming accuracy. This is applicable to basic odors and tastes for which a correct or veridical answer could be said to exist. But this does not apply to the wines and coffees, since descriptions for these refer to components of the smell and flavor profile, and there is no “correct” answer. Therefore for the wines and coffees, we calculated whether participants agreed with one another in their descriptions [21,51]. To do this, the main responses from the fully transcribed descriptions were identified. For example, a speaker gave the description for a wine displayed in Box 1.

**Box 1. Example of a Dutch wine expert’s description for the taste of Wine 4, the Vallon des Sources Vacqueyras from France.**

Em kersen in de mond. Kersen, ja amarena kersen daar gaat het naartoe. Lichte tannines, beetje bitter, maar mooi. Denk dat hij wel wat houtlaging heeft gehad maar niet overheersend.

Em, cherries in the mouth. Cherries, yes, amarena cherries that’s what it’s heading off to. Light tannins, a little bit bitter, but nice. I think he had some wood aging, but it’s not overpowering.

From this description the main qualitative descriptors *kersen* ‘cherries’, *amarena kersen* ‘amarena cherries’, *tannines* ‘tannins’, *bitter* ‘bitter’, *mooi* ‘nice’, and *houtlagering ‘*wood aging’ were coded. Modifiers and hedges were ignored unless their exclusion changed the quality description. For example, *licht* ‘light’ in *lichte tannines* ‘light tannins’ was not coded since *light* only indicates the strength of the taste (or confidence of the participant). But *amarena kersen* ‘amarena cherries’ was coded as a whole response including *amarena*, because amarena cherries may have a different quality of smell than generic cherries. Repeated responses (e.g., when a person mentioned *kersen* twice, as in the example above) were only coded once. Once the main responses were identified, the consistency between speakers was calculated using Simpson’s Diversity Index [61], a measure of diversity in a given population, or in this case, diversity of words, following Majid and Burenhult [21]. For the odor stimuli and basic tastants, where “correctness” can be determined, both agreement and accuracy were measured. Accuracy was measured by calculating the percentage of veridical answers.

## Results

### Are wines and coffees more codable for wine experts and coffee experts?

#### Length

Items that are highly codable typically receive more concise descriptions. Is this true for how wine and coffee experts describe wines and coffees? To test this, a mixed ANOVA with expertise (wine experts, coffee experts, novices) and naming task (wine smell, wine flavor, coffee smell, coffee flavor) was conducted, separately over participants (*F*_*1*_) and items (*F*_*2*_). Overall, participants had more to say about the flavors than smells of wines and coffees, *F*_*1*_(3, 180) = 22.87, *p* < .001, η_p_² = .28; *F*_*2*_(3, 16) = 34.96, *p* < .001, η_p_² = .87. In addition, wine experts talked more than novices, who in turn talked more than coffee experts, *F*_*1*_(2, 60) = 3.68, *p* = .031, η_p_² = .11; *F*_*2*_(2, 32) = 75.29, *p* < .001, η_p_² = .83. There was also an interaction between expertise and naming task, *F*_*1*_(6, 180) = 4.50, *p* < .001, η_p_² = .13; *F*_*2*_(6, 32) = 12.75, *p* < .001, η_p_² = .71. Contrary to the prediction, wine experts said more about the smell of wine (*M* = 307, *SD* = 213) than coffee experts (*M* = 156, *SD* = 136), *p* = .008, *d* = .85, but not more than novices (*M* = 232, *SD* = 203), *p* = .375. The same pattern was found for the flavor of wine: wine experts (*M* = 423, *SD* = 200) gave longer descriptions than coffee experts (*M* = 223, *SD* = 129), *p* = .001, *d* = 1.18, but their descriptions did not differ from novices (*M* = 322, *SD* = 220), *p* = .139. Turning to coffee, there were no significant differences in the length of the smell descriptions between coffee experts (*M* = 160, *SD* = 115), wine experts (*M* = 205, *SD* = 161) or novices (*M* = 215, *SD* = 185), all *ps* > .05. The same pattern was found for the flavor descriptions of coffee; again there was no difference between coffee experts (*M* = 270, *SD* = 132), wine experts (*M* = 301, *SD* = 154) or novices (*M* = 261, *SD* = 170), all *ps* > .05. So, wine experts said more about wines than the other groups, but coffee experts said the same amount as wine experts and novices about coffees, and were more succinct in general.

#### Strategy

**Fig 1:**
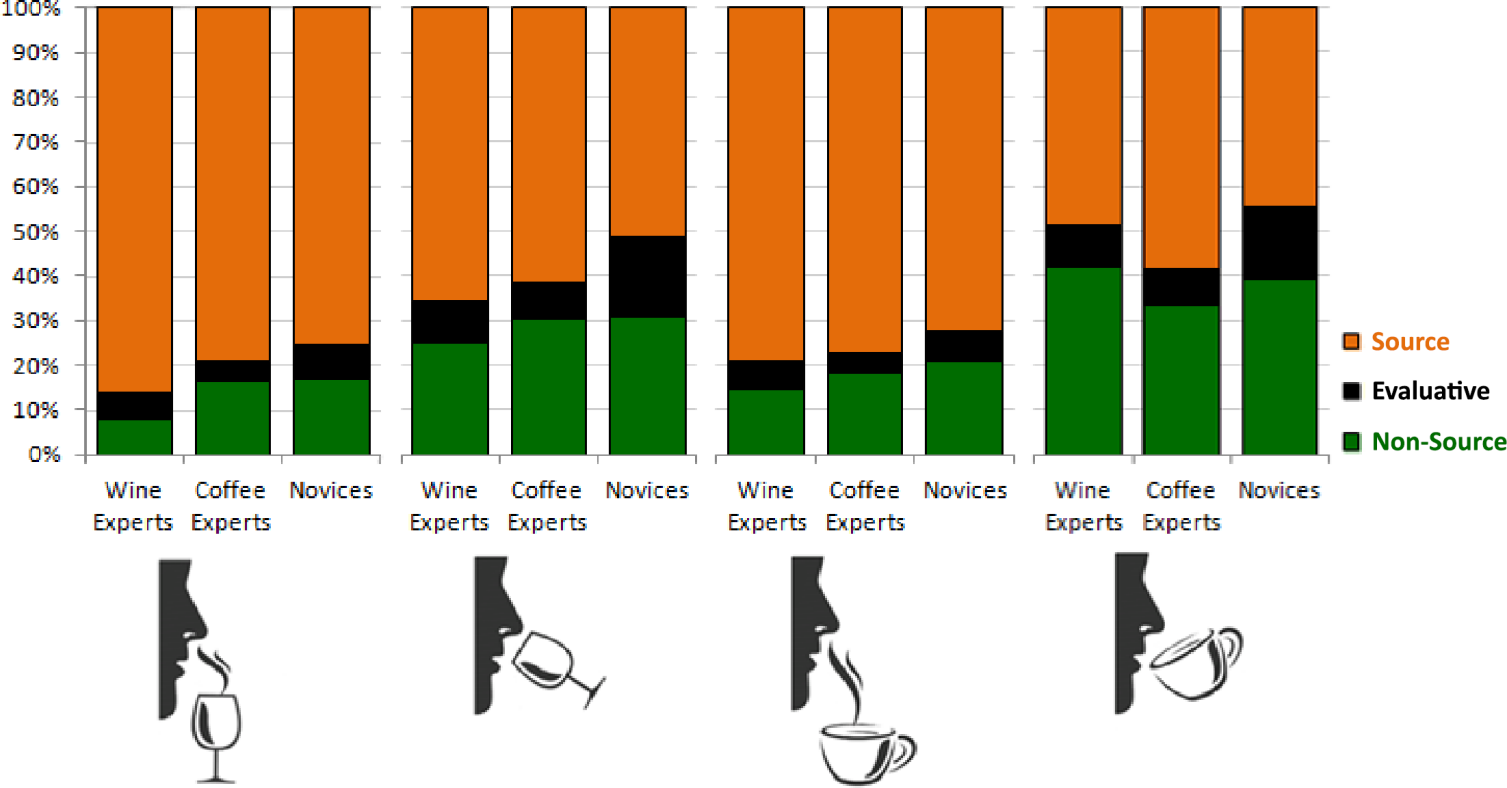
Description strategies used by wine experts, coffee experts and novices. Overall, experts and novices overwhelmingly relied on source-based descriptions (orange). However, wine experts used relatively more source-based terms to describe the smell and flavor of wine, and coffee experts used relatively more source-based terms to describe the flavor of coffee. Novices used more evaluative terms than the experts (black) to describe the smell and flavor of both coffee and wine.

Did the groups rely equally on evaluative, source-based, and non-source-based terms? The answer is no (see Fig 1). Descriptions for the smell χ^2^(4, *N* = 1115) = 21.80, *p* < .001, Cramer’s *V* = .10, and flavor χ^2^(4, *N* = 1378) = 37.80, *p* < .001, Cramer’s *V =* .12 of wine depended on expertise. Wine experts used fewer non-source-based terms (e.g., *chemisch* ‘chemical’) for wine smells *z* = -3.0, *p* = .001, while coffee experts and novices used more non-source-based terms, *z* = 1.8, *p* = .036, and *z* = 2.0, *p* = .023, respectively. Wine experts also used more source-based descriptors (e.g., *vanille* ‘vanilla’) for wine flavors *z* = 1.8, *p* = .036, and fewer non-source-based terms, *z* = -2.4, *p* = .008. Coffee experts used fewer evaluative terms for wine flavors *z* = -2.6, *p* = .005, while novices used more *z* = 3.4, *p* < .001. Novices also used fewer source-based descriptors for wine flavors *z* = -2.5, *p* = .006 than either the wine or coffee experts. So, overall, wine experts used more source-based descriptions to describe the smells and flavors of wines; coffee experts used fewer evaluative terms for wine flavor; while overall, novices used more evaluative descriptions.

For coffee smells there was no significant difference in description strategy χ^2^(4, *N* = 891) = 5.24, *p* = .263, Cramer’s *V* = .05, but there was for coffee flavor χ^2^(4, *N* = 1097) = 22.61, *p* < .001, Cramer’s *V* = .10. Just like the wine experts with wines, coffee experts gave significantly more source-based descriptors for coffees *z* = 2.0, *p* = .023. They also appeared to give fewer evaluative terms *z* = -1.6, *p* = .060, and non-source-based terms *z* = -1.6, *p* = .060. Similarly, novices gave more evaluative descriptors *z* = 2.8, *p* < .001, and fewer source terms *z* = -1.6, *p* = .060, just as they did for wines.

Overall, then, experts gave more source-based, concrete descriptions for the smells and flavors of the stimuli for which they were expert. Novices, in contrast, appeared to rely more heavily on evaluative terms, especially to describe flavors.

#### Consistency

**Fig 2:**
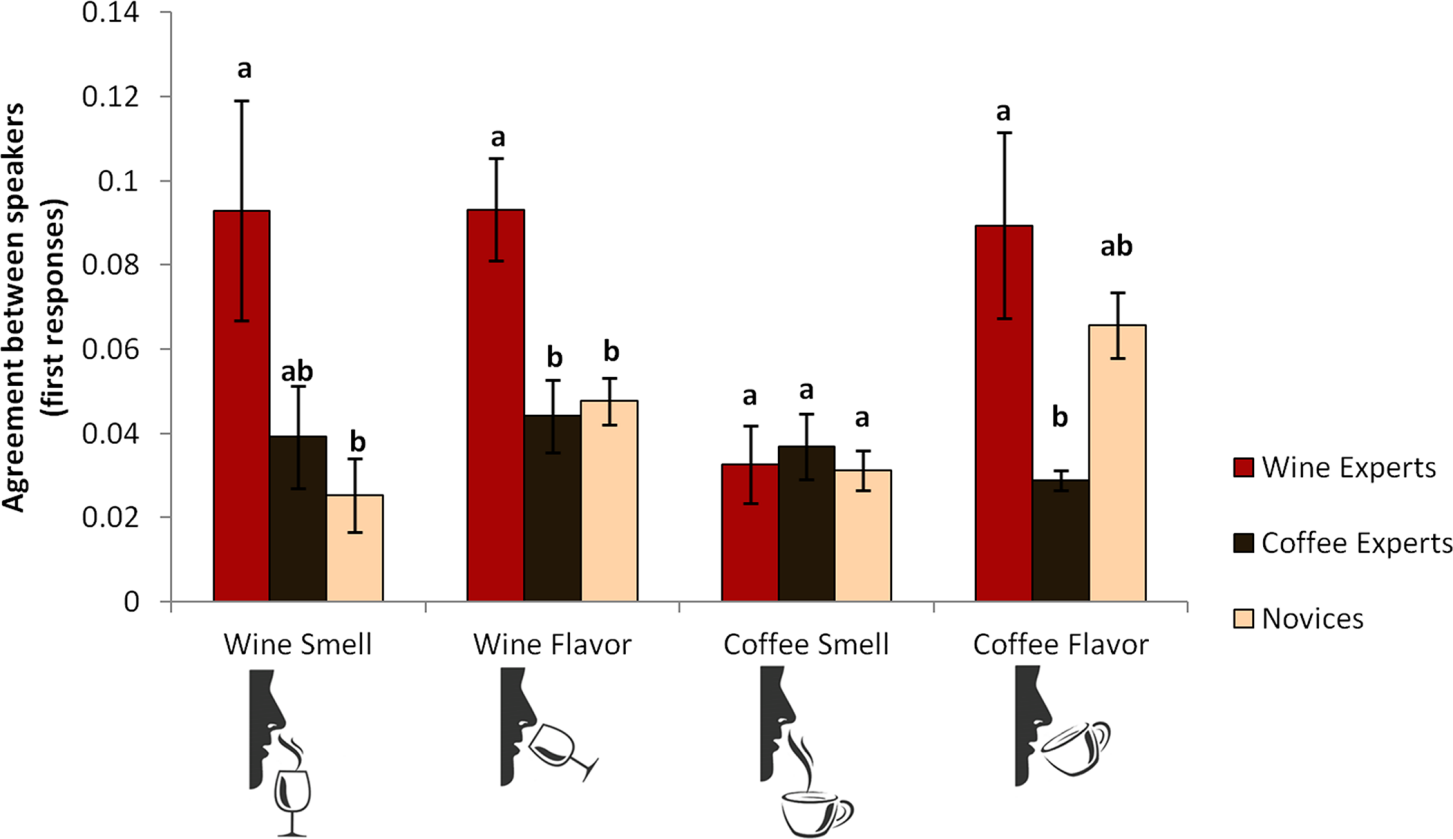
Agreement between experts and novices for wines and coffees. Wine experts were more consistent with each other in how they described the smell and flavor of wines than novices and coffee experts. In contrast, coffee experts were not more consistent than wine experts and novices for the smells and flavors of coffees. Letters indicate significant differences between groups; error bars represent ± 1 standard error.

Do experts agree with one another more in how they describe wines and coffees? To test this, an expertise (wine experts, coffee experts, novices) by naming task (wine smell, wine flavor, coffee smell, coffee flavor) mixed ANOVA was conducted using Simpson’s Diversity Index calculated over first responses. There was a main effect of expertise, showing wine experts were more consistent than coffee experts or novices, *F*(2, 12) = 17.69, *p* < .001, η_p_² = .75, and a main effect of task, with the smell and taste of wine and taste of coffee described more consistently than the smell of coffee, *F*(3, 36) = 3.27, *p* = .032, η_p_² = .21. More importantly, there was a significant interaction between expertise and naming task *F*(6, 22) = 2.76, *p* = .037, η_p_² = .43. Planned comparisons showed wine experts had higher agreement with each other when describing the smell of wine (*M* = 0.09, *SD* = 0.05) than novices (*M* = 0.03, *SD* = 0.012), *p* = .037, *d* = 1.65, but there was no significant difference between wine experts and coffee experts (*M* = 0.04, *SD* = 0.02), *p* = .112. However, when describing the flavor of wine, wine experts had higher agreement (*M* = 0.09, *SD* = 0.03) than novices (*M* = 0.05, *SD* = 0.02), *p* = .011, *d* = 1.56, and coffee experts (*M* = 0.04, *SD* = 0.02), *p* = .007, *d* = 1.96. In contrast, coffee experts did not agree more when describing the smell of coffee (*M* = 0.04, *SD* = 0.02) than novices (*M* = 0.03, *SD* = 0.01) or wine experts (*M* = 0.03, *SD* = 0.02), *p* > .05. In fact, they agreed less (*M* = 0.03, *SD* = 0.005) than the wine experts (*M* = 0.09, *SD* = 0.02) about the flavor of coffee, *p* = .025, *d* = 4.11. The results revealed no significant differences between coffee experts and novices, *p* = .237, nor between novices and wine experts, *p* = .717 for the flavor of coffee (see Fig 2). So while wine experts are more consistent in how they describe the smells and flavors of wines, coffee experts are not. This suggests expertise only has a limited role to play in linguistic codability.

The previous analysis only considered agreement on first responses. However, the analyses of description length earlier demonstrated the groups differed in the length of their descriptions. For example, wine experts described wines more elaborately than both other groups. When wine experts talk more, do they identify and name components that were identified by other experts? Or do the longer descriptions diverge more from one another? Taking all responses into account, there remained a main effect of naming task, *F*(3, 36) = 12.47, *p* < .001, η_p_² = .51, but not of expertise, *F*(2, 12) = 1.75, *p* = .215. There was an interaction between task and expertise, *F*(6, 22) = 3.19, *p* = .020, η_p_² = .47. Wine experts no longer showed more agreement on the smells of wines (*M* = 0.02, *SD* = 0.001) than coffee experts (*M* = 0.018, *SD* = 0.004), *p* = .822 or novices (*M* = 0.02, *SD* = 0.007), *p* > .05, nor did they show more agreement for the flavors of wines (*M* = 0.018, *SD* = 0.004) than coffee experts (*M* = 0.018, *SD* = 0.004), *p* > .05, or novices (*M* = 0.014, *SD* = 0.005), *p* > .05. So, talking more does not seem to increase the likelihood of converging on descriptions of smell and flavor. However, when considering all responses coffee experts showed more agreement on the smell of coffee (*M* = 0.02, *SD* = 0.003) than wine experts (*M* = 0.01, *SD* = 0.003), *p* = .033, *d* = 3.33, but not more than novices (*M* = 0.012, *SD* = 0.004), *p* = .302; nor did the novices differ from wine experts, *p* = .737. But similar to the analysis for the first responses, coffee experts agreed significantly less on the taste of coffee (*M* = 0.012, *SD* = 0.002) compared to novices (*M* = 0.025, *SD* = 0.005), *p* < .001, *d* = 3.4, and wine experts (*M* = 0.025, *SD* = 0.004), *p* = .001, *d* = 4.11.

Taken together, the results lend some support to the proposal that experts have higher codability for smells and flavors. But this agreement is rather limited in nature. Wine experts showed higher consistency when describing the smells of wines than novices, and when describing the flavor of wine and coffees than coffee experts. This suggests the wider linguistic and communicative experiences of wine experts may play a critical role for describing smells and flavors, since they perform even better than the coffee experts. However, this main effect is modulated by an interaction revealing domain-specific expertise. Wine experts agree with one another more about the smells and flavors of wines, but only when considering their first responses. When considering all responses, however, this agreement seems to disappear, possibly because each expert is isolating different components of the wine and coming to a unique linguistic profile for their experience. Coffee experts, on the other hand, only showed more agreement on the smells of coffees when taking all responses into consideration. Neither group showed a general advantage over novices across domains. So, it seems there is only a modest role of expertise when communicating about the smells and flavors of wines and coffees.

**Fig 3:**
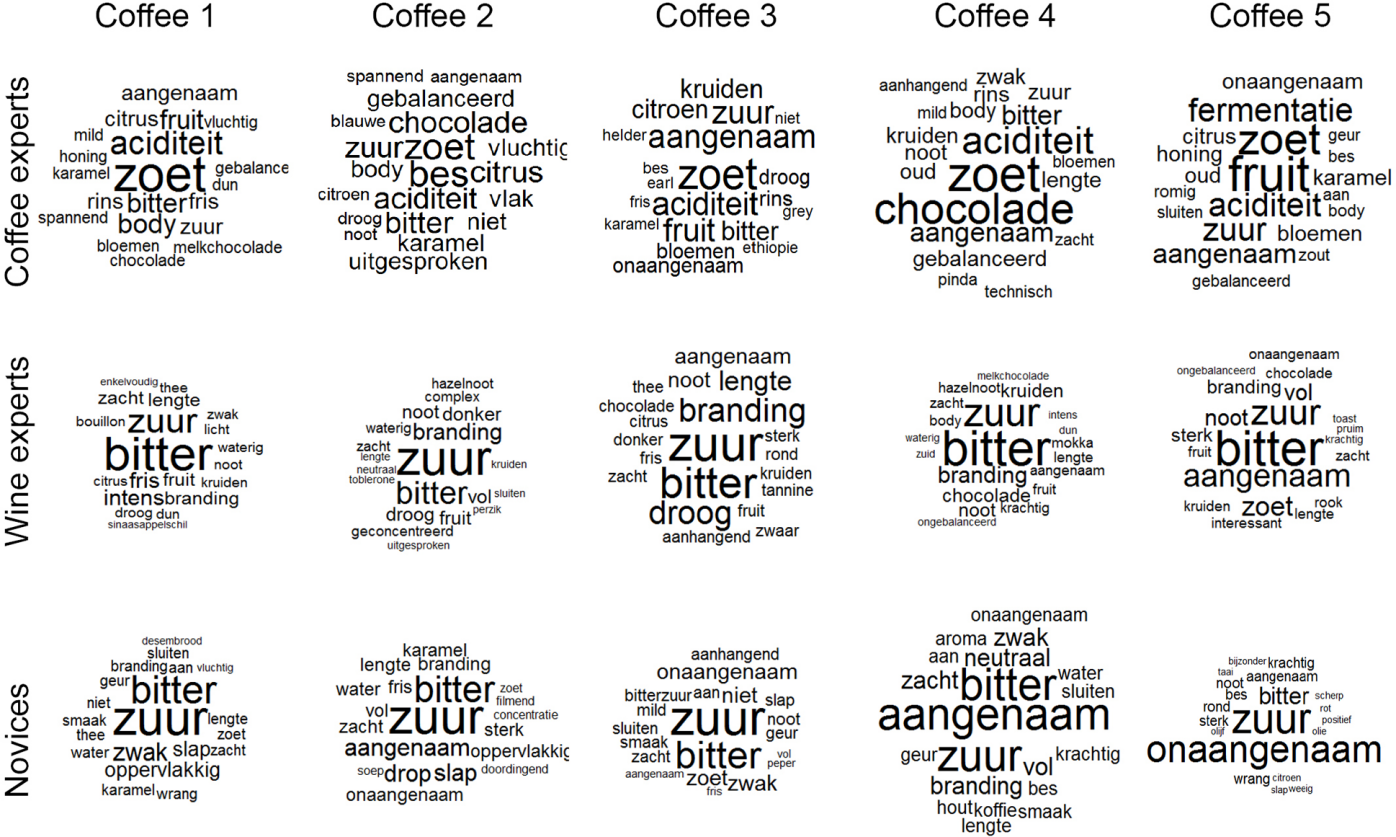
Word clouds of the 20 most frequent terms for coffee flavors. Wine experts and novices agreed more in their descriptions and predominantly describing all coffees as *bitter* and *sour*. Coffee experts, on the other hand, gave distinct flavor profiles to each coffee.

**Fig 4:**
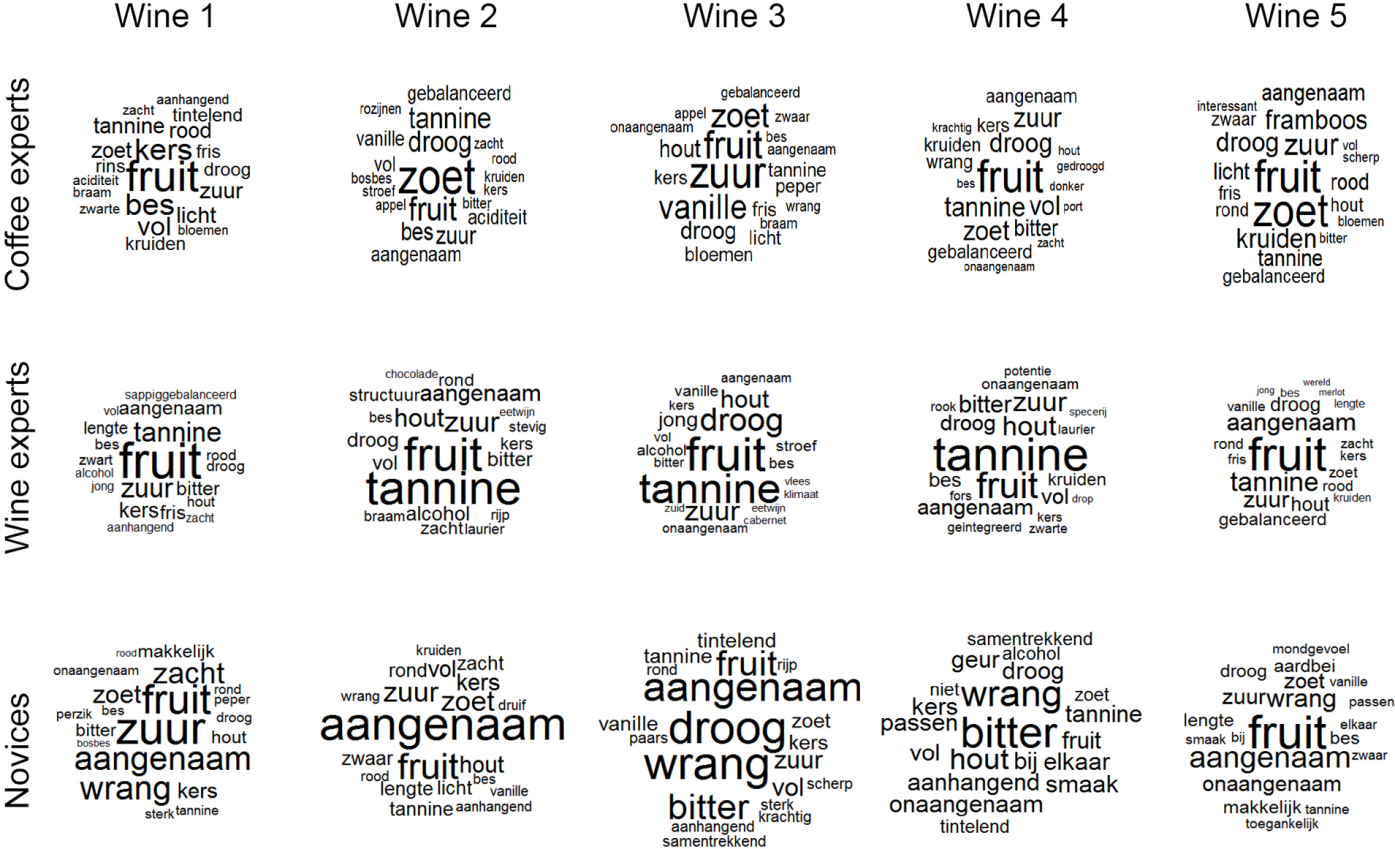
Word clouds of 20 most frequent descriptors for wine flavors. Wine experts agreed on two main qualities: *fruit* and whether the wine contained *tannins*. In addition, they identified further distinctive qualities in their descriptions. Novices commented on a number of taste qualities (e.g., *zuur* ‘sour’, *droog* ‘dry’, *wrang* ‘tart’, *bitter* ‘bitter’), and gave evaluative descriptions (e.g., *aangenaam* ‘pleasant’).

It is surprising that coffee experts show significantly less consistency for describing coffee flavors, considering describing these flavors is their core business. To better understand why this might be, we visualized the descriptions using word clouds (Fig 3 and Fig 4). In a word cloud, the relative size of a word indicates its relative frequency, with the largest words being the most frequent. The word clouds were made using the R package *wordcloud* [62]. It is clear from Fig 3 that wine experts and novices primarily described the coffees as *bitter* ‘bitter’ or *zuur* ‘sour’. And as was demonstrated by the earlier analyses, novices described items as *aangenaam* ‘pleasant’ or *onaangenaam* ‘unpleasant’. In contrast, coffee experts picked out specific flavors using source-based terms (such as *chocolade* ‘chocolate’, *bessen* ‘berries’, *kruiden* ‘herbs’). They also identified sour and bitter components, but intriguingly their most frequent taste descriptor for the same coffees was *zoet* ‘sweet’.

A comparison across the five coffees showed wine experts and novices barely distinguished between the different coffees in their descriptions, while the coffee experts identified distinct flavor profiles. For example, Coffee 4, a Brazilian Yellow Bourbon, was described by the coffee experts as ‘sweet’, ‘chocolate’, ‘balanced’, and as having ‘acidity’. This parallels the descriptors given by an independent coffee expert in a non-blind tasting: “known for its good balance between acidity, body and sweetness and for its excellent aftertaste.” [63]. Similarly, Coffee 5, a Costa Rican Villa Sarchï, was described as having ‘fruit’, ‘sweet’, and ‘acidity’, again paralleling a non-blind tasting: “Fruit acidity that’s very clean; fruit driven sweetness that’s intense.” [64].

To see whether wine experts also distinguished between the different wines, the same analysis was repeated for the flavor of wine (Fig 4). Interestingly, wine experts described the flavor of all five wines fairly similarly, by using the source-based descriptor *fruit* ‘fruit’. They also commented on the presence or absence of *tannine* ‘tannins’, noted *zuur* ‘sour’, *droog* ‘dry’, and used specific source-based descriptors, e.g. *kers*, ‘cherry’, *braam* ‘blackberry’, and *vanille* ‘vanilla’.

#### Summary

Experts used different linguistic strategies to describe their domain of expertise. Wine experts had more to say about the smell and flavor of wine, and had higher consistency in their first descriptions. Coffee experts, on the other hand, only showed higher agreement on the smells of coffees when considering their full responses. Despite these differences, both expert groups relied more on source-based descriptions to describe the stimuli from their expert domain, while novices took a more evaluative stance.

Although coffee experts did not show higher levels of agreement in their descriptions of coffee tastes, their responses appear to be more distinctive for each type of coffee than wine experts’ or novices’. In fact, their descriptions provided when blind-tasting coffees overlapped considerably with expert coffee descriptions from a non-blind tasting. This suggests although coffee experts did not show higher agreement, they nevertheless were distinctive in their linguistic descriptions. A parallel analysis of the wine experts’ descriptions of wine showed the wine experts agreed on the same two main characteristics for all the wines, and that some coffee experts and novices recognized those too, albeit to a lesser extent.

### Do experts have an advantage in naming basic smells and tastes?

To further test the domain-specificity of linguistic descriptions of smells and tastes, we tested experts and novices on simple everyday odors (e.g., cinnamon, lemon) and tastes (e.g., sweet, sour), as well. We first consider whether there was a general expertise advantage for smells and then tastes.

#### Odor naming task

Length: Do experts give more concise descriptions for smell stimuli outside their domain of expertise? A one-way ANOVA comparing the different groups on the number of characters in the descriptions showed an effect of expertise *F*_*1*_(2, 62) = 2.61, *p* = .082, η² = .08, *F*_*2*_(2, 27) = 12.71, *p* = .001, η² = .59. Coffee experts gave the shortest descriptions (*M* = 102, *SD* = 103); significantly shorter than wine experts (*M* = 146, *SD* = 125) *p* = .002, *d* = .38, and novices (*M* = 144, *SD* = 127), *p* = .012, *d* = .36. Wine experts and novices did not differ from each other, however, *p* > 0.5.

Strategy: Odors were described differently depending on expertise, χ^2^(4, *N* = 1698) = 22.90, *p* < .001, Cramer’s *V* = .08. Wine experts used more non-source-based terms *z* = 2.2, *p* = .015, while coffee experts used them less frequently *z* = -2.0, *p* = .025. In contrast, coffee experts used more source-based terms, *z* = 1.8, *p* = .036. In addition, coffee experts also used fewer evaluative terms *z* = -2.3, *p* = .010, while novices used more, *z* = 1.9, *p* = .029.

Agreement: Comparing agreement using Simpson’s Diversity Index showed no significant effect for expertise in either first *F*(2, 29) = .90, *p* = .417, η² = .06 or all responses *F*(2, 29) = 1.25, *p* = .302, η² = .09.

**Fig 5:**
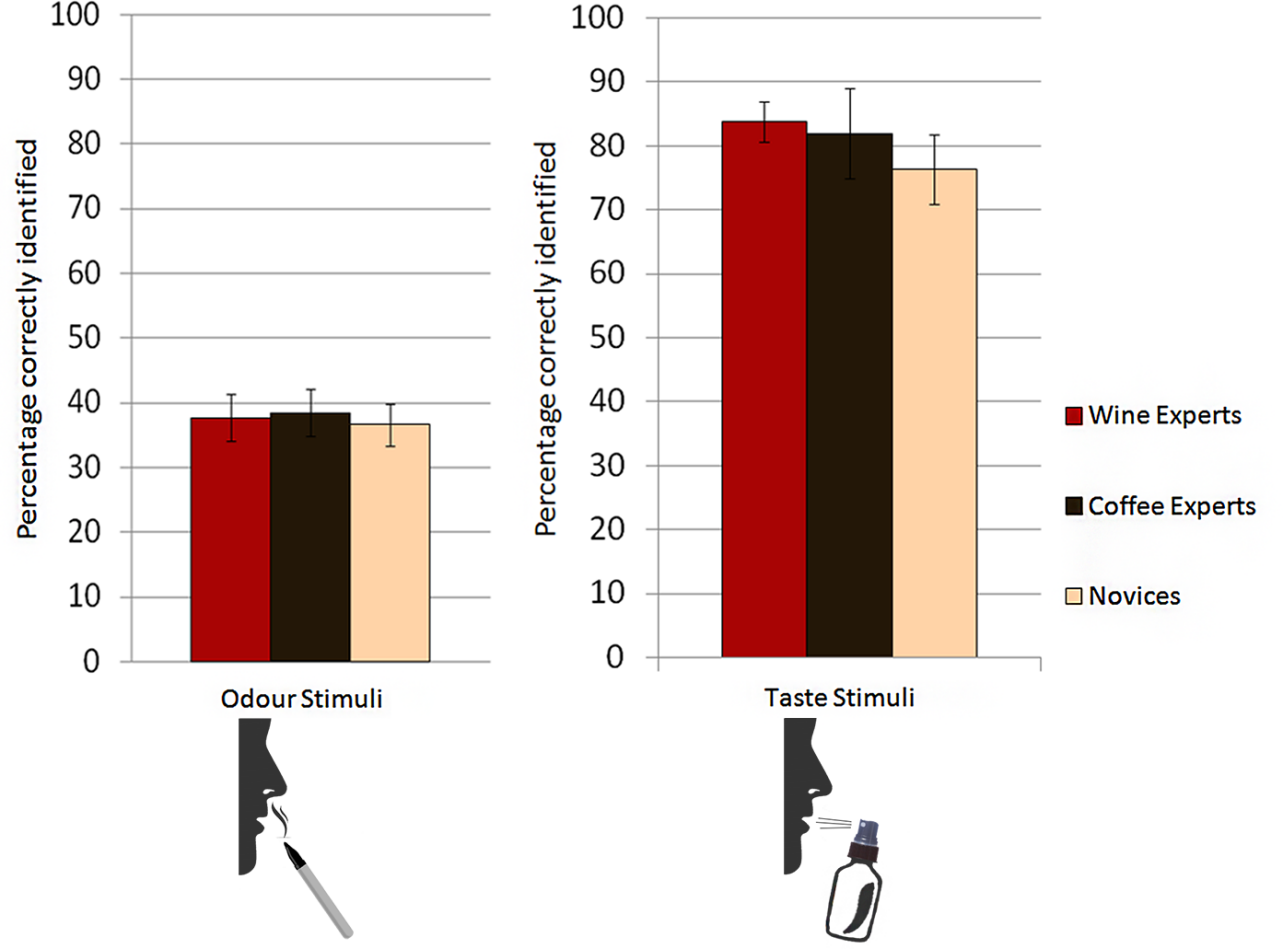
Correct responses for smell and taste stimuli. There was no significant difference between groups in the percentage of correctly named smells or tastes. Error bars represent ± 1 standard error.

Accuracy: We also compared the percentage of correct answers in the full descriptions. There was no difference between groups *F*_*1*_(2, 62) = .07, *p* = .936, η² = .01, *F*_*2*_(2, 28) = .40, *p* = .677, η² = .04 (see Fig 5).

#### Taste naming task

Length: There was a significant effect of expertise on length *F*_*1*_(2, 62) = 3.24, *p* = .046, η² = .10; *F*_*2*_(2, 14) = 24.82, *p* = .002, η² = .78. Wine experts (*M* = 113, *SD* = 18) and novices (*M* = 112, *SD* = 31) gave descriptions of the same length, *p* = .964, *d* = .01. However, coffee experts gave significantly shorter descriptions (*M* = 67, *SD* = 19) than novices, *p =* .002, *d* = 2.51, and wine experts, *p* = .003, *d* = 1.76.

Strategy: The groups differed in the linguistic strategy used to describe tastes, χ^2^(4, *N* = 1496) = 16.91, *p* = .002, Cramer’s *V* = .08. Coffee experts used significantly fewer evaluative terms *z* = -2.6, *p* = .005 than the wine experts or the novices. No other word type frequencies were statistically different from the expected model.

Agreement: There was no difference between groups in agreement in first responses, *F*(2, 23) = 1.49, *p* = .249, η² = .12. However, there was an effect of group when considering all descriptions *F*(2, 23) = 16.46, *p* < .001, η² = .61. Coffee experts agreed with one another more in how to describe basic tastes (*M* = 0.23, *SD* = 0.06) than wine experts (*M* = 0.14, *SD* = 0.04), *p* = .001, *d* = 1.77, and novices (*M* = 0.12, *SD* = 0.02), *p* < .001, *d* = 2.46, while novices and wine experts did not differ from each other, *p* = .107.

Accuracy: There was no difference between the groups in the percentage of correctly identified tastes in full descriptions *F*_*1*_(2, 62) = .54, *p* = .584, η² = .02, *F*_*2*_(2, 14) = 3.01, *p =* .082, η² = .30 (see Fig 5).

#### Summary

Overall, when describing everyday smells and basic tastes, wine experts appeared to talk the most, and coffee experts the least. Novices tended to give more evaluative responses for both smells and tastes than experts. Agreement and accuracy did not differ between groups, apart from a slight advantage for naming basic tastes by coffee experts, when all responses were considered. This may have to do with the fact that coffee experts’ are trained to seek a coffee that is the perfect balance of bitter, sour, and sweet.

## Discussion

The smell and flavor of wine and coffee seems to be described differently by wine and coffee experts in comparison to novices. Wine experts agreed more on the smell and flavor of wine, and this coincided with the use of more specific source-based terms compared to novices. Coffee experts used a similar strategy for the smell and flavor of coffee, and their descriptions were more succinct than those of novices. But this did not lead to higher agreement between the speakers for the smell and flavor of coffee. The results did not show a general influence of expertise on flavor naming. Differences in talk between wine and coffee experts, where apparent, only appear in their own domains of expertise. So, wine and coffee training only appears to play a limited role in how people talk about smells and flavors.

### Wine speak

It was unclear from the prior studies whether wine experts really were better at describing the smells and flavors of wines than non-experts. Previous studies differed in the stimuli used to test the verbal abilities of wine experts, and in the criteria used to measure those descriptions. Some studies used simple odors [40,46], while other studies used wines [35,39,44]. Some studies examined the types of terms experts use [34,35,37,39], while others took more quantitative measures, such as agreement between speakers [46]. The present study combined these qualitative and quantitative approaches, to get a better understanding of what happens when flavor experts communicate about smells and flavors. We found wine experts talked more, and used more specific source-based terms to describe the smell and taste of wine, which converges with some previous findings [33–35]. In addition, and contrary to other findings (cf. [46]), wine experts reached higher agreement than novices when describing wines.

In contrast to previous studies [28–31], we found wine experts used very few metaphors. This could be because of the specific task we used. Tasting notes on websites and in magazines written by wine experts serve an entertainment, or literary function in addition to giving information about wine. Examination of these materials tends to show an enhanced reliance on metaphor. In this experiment, participants were asked to give descriptions as precisely as possible, which did not encourage (nor discourage, particularly) metaphorical constructions. This context is comparable to how wine experts communicate during “tastings”, or when they sell wines to consumers face-to-face. In this context, experts seem to rely on more concrete vocabulary.

One notable aspect in this study was the different linguistic behavior of wine and coffee experts. This difference between groups of experts is surprising given that a previous study [40] revealed no apparent differences in smell descriptions between flavor experts. In the present study, wine experts were verbose and agreed on the descriptions for wine; the coffee experts were overall more succinct. These differences in descriptions in the present study are unlikely to be caused by intrinsic properties of the stimuli, as the wines and coffees were sorted in comparable ways by novice participants in a control study. Both groups were also comparable in amount of expertise. Wine and coffee experts were both professionals, earning their living with their knowledge. These criteria were independently confirmed by the expertise questionnaires. Moreover, the odor awareness questionnaire showed both expert groups were equally aware of odors in daily life (and more so than the novices). So the differences between expert groups are unlikely to be due to these factors.

Instead we suggest wine experts differ from coffee experts because of the different language games surrounding these two industries. While “wine talk” is an attested genre, there is little comparable “coffee talk” (i.e., about coffee, rather than over coffee). As we suggested in the introduction, wine experts have more opportunities to read, listen, and talk about the smells and flavors of wines (e.g., in magazines, menus, tastings, etc.), than coffee experts do for coffees. This means the two expert groups are doing different things when communicating about smells and flavors in their daily life. As Silverstein [65] suggests, wine experts are arguably indexing how much they know about the wines, as much as they are describing the properties of the wine itself.

### Codability

We had asked whether smells and flavors were linguistically expressed more easily by experts than novices. Linguistic expressibility is a complex notion that can be operationalized in various ways (cf. [13]). We focused on length of description, types of responses, agreement between speakers and accuracy, following the classic work of Brown and Lenneberg [21,51]. They [51] asked English speakers to name colors and found exactly those colors with concise descriptions also had short reaction times, and within- and across-speaker agreement. They then derived a single composite measure of linguistic “codability”, combining these measures, and found color chips with high codability were also remembered better. This suggests differential linguistic coding can have wider impact on memory and perception, a proposal that has recently found further support in the domain of color, for example [66–69].

Our results did not show the same alignment of length and agreement found in these earlier studies. Wine experts had higher agreement yet gave longer descriptions, while coffee experts gave short descriptions but did not agree. So, perhaps this way of examining the linguistic behavior of experts needs to be reconsidered. It seems as if length is not a diagnostic measure in this study, since longer talk appears to index the speaker’s orientation, rather than indicate how difficult the entity was to describe. More importantly, earlier studies (which have found length to coincide with agreement) have asked speakers to name stimuli, rather than describe them. In sum this suggests agreement is likely the more informative measure in our study. On this measure we find a small advantage for experts when describing stimuli from their own domain of expertise.

Across the board, people tended to use source-based descriptions (e.g., *berry*, *vanilla)*, but both expert groups tended to use more such descriptions in their domain of expertise. It appears that expert descriptions may be more informative. Compare a coffee expert’s descriptions for coffee number five—e.g., “a fruity, acidic coffee with a fermented aroma and hints of caramel, honey and citrus”—with a novice’s—e.g., “a sour and unpleasant coffee with some hints of berry”, for example. In order to verify this, future studies could also examine whether people find it easier to understand expert descriptions than novices’, by conducting a director-matcher task, where people have to match wines and coffees to descriptions (cf. [38]). Some previous work, indeed, suggests descriptions from experts are better matched to the original stimulus than those produced by novices [35, 44, 70]. Our current results indicate there may be differences depending on the expert and the domain. It would be interesting to examine whether wine and especially coffee expert descriptions are equally informative when given to other experts or novices.

Finally, prior research in other domains (e.g., color) shows a tight link between linguistic coding and memory, which raises the question whether expert memory might also be linguistically mediated. Some studies have found wine experts’ recognition memory to be superior to that of novices’ [37,39,46,71], although a link between experts’ language use and recognition memory has not been reliably demonstrated. This is a matter for future research.

### Culture and sub-culture

For wine and coffee experts, smells and flavors play an important role in their daily routine, and experts can be seen as part of a sub-culture, with specific practices revolving around smell and flavor [25]. One explanation for the finding wine experts are better at describing the smells and flavors of wine is that wine experts often engage in talk about wine (cf. [26]), which trains them use language in a specific way. This suggests that to become better at describing smells and flavors, not only is it important to have abundant perceptual experience (cf. [52, 72]), but also to train verbalizing these experiences.

Yet another possibility to explain the differences between wine and coffee experts lies in the way these experts appreciate wine and coffee, respectively. During a normal wine tasting or judgment session, wine experts first note the color, before the wine is smelled (cf. [25]). Smelling is sometimes composed of two parts, where the wine is first smelled when it rests still in the glass, and second when the glass is swirled to release additional aromas. Flavor appreciation comes after this, where among other things, experts pay attention to how sweet or dry a wine is, what mouthfeel it produces, and how long the aftertaste lingers.

Coffee experts approach coffee judgments during cupping in a slightly different manner. As with the wine experts, coffee experts first note the color of the ground coffee. But for the coffee experts, the smelling component of cupping is divided into three parts: first the dry, freshly ground coffee is smelled (the so-called “fragrance of the coffee”). Water is then poured on the coffee. The “crust” that has formed on top of the coffee is then “broken” by stirring it gently with a spoon. The aroma of the coffee is smelled at this stage too. Finally, after the coffee has steeped for a while, the aroma of the coffee is judged a final time. The three orthonasal parts are combined in a single aroma quality judgment. The coffee is then tasted from a spoon, to get as much air as possible with the coffee sample in the mouth. During this stage, coffee experts, similar to wine experts, pay attention to how sweet the coffee is, what (retronasal) flavors are in the sample, etc. [54]. In the present study, to make the tasks and subsequent data more comparable across the two domains, participants were only able to smell the coffee when it had already steeped for some time. It could be the case that coffee experts would achieve higher agreement were they to smell and describe during these other phases. Future studies specifically investigating coffee expertise are required in order to test coffee experts’ abilities to describe the various aspects of orthonasal coffee olfaction.

Overall, however, the main expert advantage we found was when wine experts described stimuli from their own domain of expertise. In contrast, the Jahai are better in describing smells regardless the domain or category the smell comes from [21]. An indirect comparison of the present study to the study by Majid and Burenhult [21] appears to indicate Jahai speakers have higher codability for smells they have never encountered before than wine experts have for smells from sources encountered every day. Even after many years of experience, experts do not appear to show the linguistic prowess for smells the Jahai have. Why might this be so?

There are at least two possible explanations. First, there may be some genetic difference between Jahai speakers and Western speakers that enables the Jahai to talk about smells with relative ease. There are wide-spread differences between populations in olfactory genes [73,74], and different sensitivity for specific odorants [75]. In addition, populations differ in olfactory discrimination [76–78].

A second possibility has to do with the age of acquisition of smell and flavor vocabularies. Children with different cultural backgrounds are socialized in different ways with regard to the senses, and in some communities children are taught smell is an important part of the world (cf. [79]). In particular, Jahai speakers learn smell vocabulary as children as part of normal language acquisition, unlike wine and coffee experts. Training for experts does not begin until they are adults, long past any critical period for language acquisition. It could be the wine and coffee experts simply cannot overcome this maturational limitation.

## Conclusion

In sum, it appears sensory experience and cultural preoccupation alone is not enough to overcome the boundaries of language. Wine and coffee experts have only a small advantage over novices when describing smells and flavors, limited to their domain of expertise. We suggest more emphasis needs to be given to the verbal practices around smells and flavors, in addition to aspects surrounding expert perceptual training. After all, in order to decide what wine or coffee to buy, or to choose a food and drink pairing, or simply to convey our aesthetic appreciation, we use language. Our perceptual experiences are shared through our common tongue.

To conclude, perceptual experience alone is not enough to overcome the boundaries of language; verbal training is also essential in order to effectively communicate about smells and flavors.

## References

[1] Small DM, Prescott J. Odor/taste integration and the perception of flavor. Exp Brain Res. 2005; 166: 345–357. 16028032 10.1007/s00221-005-2376-9

[2] Spence C. Multisensory flavor perception. Cell. 2015; 161: 24–35. 10.1016/j.cell.2015.03.007 25815982

[3] Cain WS. To know with the nose: Keys to odor identification. Science. 1979; 203: 467–470. 760202 10.1126/science.760202

[4] Engen T. Remembering odors and their names. Am Sci. 1987; 5: 497–503.

[5] Yeshurun Y, Sobel N. An odor is not worth a thousand words: From multidimensional odors to unidimensional odor objects. Annu Rev Psychol. 2010; 61: 219–241. 10.1146/annurev.psych.60.110707.163639 19958179

[6] Olofsson JK, Gottfried JA. The muted sense: Neurocognitive limitations of olfactory language. Trends Cogn Sci. 2015; 19: 314–321. 10.1016/j.tics.2015.04.007 25979848 PMC4457599

[7] Horverak Ø. Wine journalism-Marketing or consumers' guide? Market Sci. 2009; 28: 573–579.

[8] Gluck M. Wine language: Useful idiom or idiot-speak In: Atchinson J, Lewis D, editors. New media language 2003: 107–115.

[9] Quandt RE. On wine bullshit: Some new software? Journal of Wine Economics. 2007; 2: 129–135.

[10] Weil RL. Debunking critics' wine words: Can amateurs distinguish the smell of asphalt from the taste of cherries? Journal of Wine Economics. 2007; 2: 136–144.

[11] Myers CS. The taste‐names of primitive peoples. Brit J Psychol 1904; 1: 117–126.

[12] Sperber D. Rethinking symbolism English translation by Morton AL. Cambridge: Cambridge University Press; 1975 p. 172

[13] Levinson SC, Majid A. Differential ineffability and the senses. Mind Lang. 2014; 29: 407–427.

[14] San Roque L, Kendrick KH, Norcliffe E, Brown P, Defina R, Dingemanse M, et al Vision verbs dominate in conversation across cultures, but the ranking of non-visual verbs varies. Cog Ling. 2015; 26: 31–60.

[15] Cain WS, de Wijk R, Lulejian C, Schiet F, See LC. Odor identification: Perceptual and semantic dimensions. Chem Sens. 1998; 23: 309–326. 10.1093/chemse/23.3.309 9669044

[16] Lawless H, Engen T. Associations to odors: Interference, mnemonics, and verbal labeling. J Exp Psychol Learn Mem Cogn. 1977; 3: 52. 845551

[17] McAuliffe WK, Meiselman HL. The roles of practice and correction in the categorization of sour and bitter taste qualities. Percept Psychophys. 1974; 16: 242–244.

[18] O'Mahony M, Goldenberg M, Stedmon J, Alford J. Confusion in the use of the taste adjectives ‘sour’ and ‘bitter’. Chem Sens. 1979; 4: 301–318.

[19] O'Mahony M, Ishii R. A comparison of English and Japanese taste languages: Taste descriptive methodology, codability and the umami taste. Brit J Psychol. 1986; 77: 161–174. 3730725 10.1111/j.2044-8295.1986.tb01991.x

[20] Majid A. Cultural factors shape olfactory. Trends Cogn Sci. 2015; 19: 629–630 10.1016/j.tics.2015.06.009 26440119

[21] Majid A, Burenhult N. Odors are expressible in language, as long as you speak the right language. Cognition. 2014; 130: 266–270. 10.1016/j.cognition.2013.11.004 24355816

[22] Burenhult N, Majid A. Olfaction in Aslian ideology and language. Senses & Society. 2011; 6: 19–29.

[23] Wnuk E, Majid A. Revisiting the limits of language: The odor lexicon of Maniq. Cognition. 2014; 131: 125–138. 10.1016/j.cognition.2013.12.008 24462926

[24] Henrich J, Heine SJ, Norenzayan A. The weirdest people in the world? Behav Brain Sci. 2010; 33: 61–83. 10.1017/S0140525X0999152X 20550733

[25] Herdenstam AP, Hammarén M, Ahlström R, Wiktorsson PA. The professional language of wine: Perception, training and dialogue. J Wine Res. 2009; 20: 53–84.

[26] Silverstein M. “Cultural” concepts and the language‐culture nexus. Curr Anthropol. 2004; 45: 621–652.

[27] Ortega-Heras M, González-SanJosé ML, Beltrán S. Aroma composition of wine studied by different extraction methods. Anal Chim Acta. 2002; 458: 85–93.

[28] Suárez Toste E. Metaphor inside the wine cellar: On the ubiquity of personification schemas in winespeak. Metaphorik. 2007; 12: 53–64.

[29] Caballero R, Suárez-Toste E. A genre approach to imagery in winespeak: Issues and prospects. In: Low G, Todd Z, Deignan A, Cameron L, editors. Researching and applying metaphor in the real world. 2010; 26: p. 265.

[30] Paradis C, Eeg-Olofsson M. Describing sensory experience: The genre of wine reviews. Metaphor Symb. 2013; 28: 22–40.

[31] Wipf B. Wine writing meets MIPVU: Linguistic metaphor identification of wine notes (M. Sc. Thesis). Amsterdam: VU University; 2010.

[32] Caballero R. Manner-of-motion verbs in wine description. J Pragmat. 2007; 39: 2095–2114.

[33] Chollet S, Valentin D. Expertise level and odour perception: What can we learn from red burgundy wines? Année Psychol. 2000; 100: 11–36. French.

[34] Lawless HT. Flavor description of white wine by “expert” and nonexpert wine consumers. J Food Sci. 1984; 49: 120–123.

[35] Solomon GE. Psychology of novice and expert wine talk. Am J Psychol. 1990; 103: 495–517.

[36] Solomon GE. Conceptual change and wine expertise. J Learn Sci. 1997; 6: 41–60.

[37] Zucco GM, Carassai A, Baroni MR, Stevenson RJ. Labeling, identification, and recognition of wine-relevant odorants in expert sommeliers, intermediates, and untrained wine drinkers. Perception. 2011; 40: 598–607. 21882722 10.1068/p6972

[38] Lehrer A. Wine and conversation Oxford: Oxford University Press; 1983.

[39] Melcher JM, Schooler JW. The misremembrance of wines past: Verbal and perceptual expertise differentially mediate verbal overshadowing of taste memory. J Mem Lang. 1996; 35: 231–245.

[40] Sezille C, Fournel A, Rouby C, Rinck F, Bensafi M. Hedonic appreciation and verbal description of pleasant and unpleasant odors in untrained, trainee cooks, flavorists, and perfumers. Front Psychol. 2014; 5: 12 10.3389/fpsyg.2014.00012 24478743 PMC3900918

[41] Bende M, Nordin S. Perceptual learning in olfaction: Professional wine tasters versus controls. Physiol Behav. 1997; 62: 1065–1070. 9333201 10.1016/s0031-9384(97)00251-5

[42] Lehrer A. Talking about wine. Language. 1975; 51: 901–923.

[43] Tempere S, Hamtat ML, Revel G, Sicard G. Comparison of the ability of wine experts and novices to identify odorant signals: A new insight in wine expertise. Aust J Grape Wine Res. 2015 10.1111/ajgw.12192

[44] Gawel R. The use of language by trained and untrained experienced wine tasters. J Sens Stud. 1997; 12: 267–284.

[45] Smith BC. The objectivity of taste and tasting In: Smith BC, editor. Questions of taste: The philosophy of wine. Oxford: Signal Books Limited; 2007 p. 41–98.

[46] Parr WV, Heatherbell D, White KG. Demystifying wine expertise: Olfactory threshold, perceptual skill and semantic memory in expert and novice wine judges. Chem Sens. 2002; 27: 747–755. 10.1093/chemse/27.8.747 12379599

[47] Royet JP, Plailly J, Saive AL, Veyrac A, Delon-Martin C. The impact of expertise in olfaction. Front Psychol. 2013; 4: 928 10.3389/fpsyg.2013.00928 24379793 PMC3861696

[48] Grosch W. Chemistry III: Volatile compounds In: Clarke R, Vitzthum OG, editors. Coffee: Recent developments. Oxford: Blackwell Science Ltd; 2001: p. 68–89.

[49] Shibamoto T. An overview of coffee aroma and flavor chemistry In: Quatorzieme colloque scientifique international sur le cafe. San Francisco; International Scientific Colloquium on Coffee 1991 p. 107–116.

[50] Lev‐Ari S. How the size of our social network influences our semantic skills. Cognitive Sci. 2015 10.1111/cogs.12317 26515021

[51] Brown RW, Lenneberg EH. A study in language and cognition. J Abnorm Soc Psychol. 1954; 49: 454. 10.1037/h0057814 13174309

[52] Hughson AL, Boakes RA. Perceptual and cognitive aspects of wine expertise. Aust J Psychol. 2001; 53: 103–108.

[53] Smeets MA, Schifferstein HN, Boelema SR, Lensvelt-Mulders G. The Odor Awareness Scale: A new scale for measuring positive and negative odor awareness. Chem Sens. 2008; 33: 725–734. 10.1093/chemse/bjn038 18622009

[54] Specialty Coffee Association of America (Internet). (cited 2015 Apr 4). Available: http://www.scaa.org/?page=resources&d=cupping-protocols

[55] Pagès J. Collection and analysis of perceived product inter-distances using multiple factor analysis: Application to the study of 10 white wines from the Loire Valley. Food Qual Prefer. 2005; 16: 642–649.

[56] Husson F, Josse J, LeDien S, Pages J. FactoMineR: Exploratory Multivariate Data Analysis with R (Internet). (cited 2016 Jan 7). Available: http://factominer.free.fr/

[57] Hummel T, Sekinger B, Wolf SR, Pauli E, Kobal G. ‘Sniffin’sticks': Olfactory performance assessed by the combined testing of odor identification, odor discrimination and olfactory threshold. Chem Sens. 1997; 22: 39–52. 10.1093/chemse/22.1.39 9056084

[58] Prescott J. Comparisons of taste perceptions and preferences of Japanese and Australian consumers: Overview and implications for cross-cultural sensory research. Food Qual Prefer. 1998; 9: 393–402.

[59] Robinson JO. The misuse of taste names by untrained observers. Br J Psychol. 1970; 61: 375–378. 5457507 10.1111/j.2044-8295.1970.tb01254.x

[60] Hummel T, Kobal G, Gudziol H, Mackay-Sim A. Normative data for the “Sniffin’Sticks” including tests of odor identification, odor discrimination, and olfactory thresholds: An upgrade based on a group of more than 3,000 subjects. Eur Arch Oto-Rhino-L. 2007; 264: 237–243. 10.1007/s00405-006-0173-0 17021776

[61] Simpson EH. Measurement of diversity. Nature. 1949; 163, p. 688.

[62] Fellows I. wordcloud « Fells Stats (Internet). 2013 (cited 2016 Jan 7). Available: http://blog.fellstat.com/?cat=11

[63] Coffee Origins of the world (Internet). (cited 2015 Jun 9). Available: http://www.specialtycoffee.nl/en/coffee/origins

[64] Has Bean Coffee—Villa Sarchi (Internet). (cited 2015 Jun 29). Available: http://www.hasbean.co.uk/blogs/varietals/15254989-villa-sarchi

[65] Silverstein M. Old wine, new ethnographic lexicography. Annu Rev Anthropol. 2006; 35: 481–96.

[66] Mitterer H, Horschig JM, Müsseler J, Majid A. The influence of memory on perception: It’s not what things look like, it’s what you call them. J Exp Psychol Learn Mem Cogn. 2009; 35: 1557 10.1037/a0017019 19857025

[67] Winawer J, Witthoft N, Frank MC, Wu L, Wade AR, Boroditsky L. Russian blues reveal effects of language on color discrimination. Proc Natl Acad Sci USA. 2007; 104: 7780–7785. 17470790 10.1073/pnas.0701644104 PMC1876524

[68] Regier T, Kay P. Language, thought, and color: Whorf was half right. Trends Cogn Sci. 2009; 13: 439–446. 10.1016/j.tics.2009.07.001 19716754

[69] Davidoff J, Davies I, Roberson D. Colour categories in a stone-age tribe. Nature. 1999; 398: 203–204. 10094043 10.1038/18335

[70] Chollet S, Valentin D. Impact of training on beer flavor perception and description: Are trained and untrained subjects really different? J Sens Stud. 2001; 16: 601–618.

[71] Parr WV, White KG, Heatherbell DA. Exploring the nature of wine expertise: What underlies wine experts' olfactory recognition memory advantage? Food Qual Prefer. 2004; 15: 411–420.

[72] Kellman PJ, Massey CM. Perceptual Learning, cognition, and expertise. The psychology of learning and motivation. 2013; 58: 117–165.

[73] Gilad Y, Lancet D. Population differences in the human functional olfactory repertoire. Mol Biol Evol. 2003; 20: 307–314. 12644552 10.1093/molbev/msg013

[74] Menashe I, Man O, Lancet D, Gilad Y. Different noses for different people. Nat Genet. 2003; 34: 143–144. 12730696 10.1038/ng1160

[75] Keller A, Zhuang H, Chi Q, Vosshall LB, Matsunami H. Genetic variation in a human odorant receptor alters odour perception. Nature. 2007; 449: 468–472. 17873857 10.1038/nature06162

[76] Sorokowska A, Sorokowski P, Frackowiak T. Determinants of human olfactory performance: A cross-cultural study. Sci Total Environ. 2015; 506: 196–200. 10.1016/j.scitotenv.2014.11.027 25460952

[77] Sorokowska A, Sorokowski P, Hummel T. Cross-cultural administration of an odor discrimination test. Chemosens Percept. 2014; 7: 85–90. 24883170 10.1007/s12078-014-9169-0 PMC4037584

[78] Sorokowska A, Sorokowski P, Hummel T, Huanca T. Olfaction and environment: Tsimane’ of Bolivian rainforest have lower threshold of odor detection than industrialized German people. PLOS ONE. 2013; 8: e69203 10.1371/journal.pone.0069203 23922693 PMC3726727

[79] Classen C. Other ways to wisdom: Learning through the senses across cultures. Int Rev Educ. 1999; 45: 269–280.

